# Nano Polymeric Carrier Fabrication Technologies for Advanced Antitumor Therapy

**DOI:** 10.1155/2013/305089

**Published:** 2013-12-04

**Authors:** Wei Li, Mengxin Zhao, Changhong Ke, Ge Zhang, Li Zhang, Huafei Li, Fulei Zhang, Yun Sun, Jianxin Dai, Hao Wang, Yajun Guo

**Affiliations:** ^1^International Joint Cancer Institute, The Second Military Medical University, 800 Xiangyin Road, Shanghai 200433, China; ^2^State Key Laboratory of Antibody Medicine and Targeting Therapy and Shanghai Key Laboratory of Cell Engineering, Shanghai 201203, China; ^3^PLA General Hospital Cancer Center, PLA Graduate School of Medicine, Beijing 100853, China; ^4^College of Pharmacy, Liaocheng University, 1 Hunan Road, Liaocheng, Shandong 25000, China; ^5^Department of Chemistry, Jinan University, Guangzhou 510632, China

## Abstract

Comparing with the traditional therapeutic methods, newly developed cancer therapy based on the nanoparticulates attracted extensively interest due to its unique advantages. However, there are still some drawbacks such as the unfavorable *in vivo* performance for nanomedicine and undesirable tumor escape from the immunotherapy. While as we know that the *in vivo* performance strongly depended on the nanocarrier structural properties, thus, the big gap between *in vitro* and *in vivo* can be overcome by nanocarrier's structural tailoring by fine chemical design and microstructural tuning. In addition, this fine nanocarrier's engineering can also provide practical solution to solve the problems in traditional cancer immunotherapy. In this paper, we review the latest development in nanomedicine, cancer therapy, and nanoimmunotherapy. We then give an explanation why fine nanocanrrie's engineering with special focus on the unique pathology of tumor microenvironments and properties of immunocells can obviously promote the *in vivo* performance and improve the therapeutic index of nanoimmunotherapy.

## 1. Introduction

Cancer, which is a leading cause of death worldwide, can be initiated by various factors such as radiation, bacterial infection, and genetic abnormalities. Today, deaths from cancer account for about one in eight deaths worldwide. This figure is projected to continue rising to an estimated 13.2 million in 2030 [1]. Traditional cancer therapies, including surgery, radiotherapy, and chemotherapy, have made significant progress in cancer therapy. However, they still cause serious side effects or death resulting from damage to normal cells and organs. The highly specific medical intervention at the molecular scale for curing disease or repairing damaged tissues, such as bone, muscle, or nerve is called “nanomedicines” as defined by National Institutes of Health in USA (https://commonfund.nih.gov/nanomedicine/overview.aspx). Evidence has shown that the cancer therapeutic index can be significantly improved with nanomedicines [2]. The *in vitro/vivo* performance of nanomedicines strongly depends on the material, size, and surface properties of the nanocarriers. The application of nanomedicines in cancer therapy overcomes the drawbacks of small therapeutic agents including poor solubility, unfavorable pharmacokinetics, low intratumoral accumulation, quick degradation, and wide tissue distribution [3, 4]. Currently, two representative nanomedicines, Doxil and Abraxane, have been approved by the U.S. Food and Drug Administration. Doxil (the trade name for the generic chemotherapy drug doxorubicin liposomes) was approved in 2003 for the treatment of ovarian cancer and multiple myeloma. Abraxane (the trade name of albumin-based nanoparticles) was approved in 2005 for the treatment of recurrent or metastatically advanced breast cancer. Many novel nanomedicine formulations based on polymeric nanoparticles, micelles, and liposomes have been extensively investigated recently for their effectiveness in tumor imaging and delivery. These nanomedicines have thus been undergone preclinical and clinical trials that have shown the high potential of nanomedicines in cancer therapy [4].

In the clinics, a tumor frequently experiences relapse, which results in therapeutic failure and an unfavorable postoperative life. This phenomenon is partly attributed to the micrometastases of disseminated cancer cells. Overcoming such a tumor relapse is critical in clinical oncology for curing tumors. Fortunately, it is known that the host immune system can recognize, eliminate, and protect the body from viral or bacterial infections as well as the extension of transformed cells (including precancer cells) [5]. Developments in the field of immunology have successfully promoted various disciplines with a special emphasis in oncology [6]. The application of immunological disciplinary in cancer therapy is termed cancer immunotherapy, which has offered new hopes for more efficient cancer treatment and started from the late nineteenth century. Now, cancer immunotherapy mainly refers to approaches that modify the host immune system and/or utilize the components of immune system for cancer treatment. Over the last 25 years, 17 immunotherapeutic products have been approved for cancer treatment. Among them, cancer vaccines play a vital important role [7–10]. Two prophylactic HBV/HPV (hepatitis B virus vaccines and human papillomavirus) and one therapeutic cancer vaccines (Provenge) have been approved by FDA [11, 12]. A virus-ike particle-based vaccine (VLP), Gardasil, has generated over €3 billion in revenue in the market since 2009 [2, 13, 14]. Immunotherapy has become increasingly attractive because not only can it kill primary tumor cells but also instruct the immune system to eradicate the disseminated tumor cells/micrometastasis in the blood circulation and distant organs. Herein, this paper illustrates the state-of-the-art development in nanomedicine and cancer immunotherapy. The finely micellar structure tailoring for promoting its *in vivo/vitro* application was discussed. We further illustrate how to promote the nanoimmunotherapy by the chemical design and finely carrier's engineering with special focus on the unique pathology of tumor microenvironments and properties of immunocells.

## 2. Finely Assembled Micelles for Promoting Antitumor Therapy

Almost 40% of newly discovered drugs have delivery problems due to their low solubility, permeability, and stability [15]. In comparison with the traditional small molecule therapeutic agent, nanomedicine has offered new hope for detection, prevention, and treatment in cancer therapy because it extensively improves the solubility of poorly water-soluble drugs [16], prolongs the half-life of drug systemic circulation [17], releases drugs at a controlled rate [18], delivers drugs in a targeted manner with little side effects, suppresses drug resistance, and reduces the immunogenicity [16]. Nanomedicine was generally defined as use and development of nanoscale or nanostructured materials to solve the problems in medicine via its unique medical effects (https://commonfund.nih.gov/nanomedicine/overview.aspx). With the rapid advances in nanotechnology, many cancer therapeutic agents delivering systems have been developed based on nanoparticles such as polymeric micelles, polymer-drug conjugates, dendrimers, liposomes, nanopolymer composition, and inorganic particulates with a size range of 1–1,000 nm. Some of these products have been introduced into the pharmaceutical market. Doxil was the first liposomal drug formulation for the treatment of AIDS associated with Kaposi's sarcoma in 1995 [19]. The polymer-drug conjugate, Abraxane, an albumin-bound paclitaxel drug formulation, was approved by the Food and Drug Administration, USA (FDA) in 2005 as a second-line treatment for the breast cancer [20–22].

However, some major challenges are raised as the clinical test of numerous ensuing nanomedicine products. The obvious drawbacks are the *in vivo* instability [23] and the fast clearance from the blood by the reticuloendothelial system (RES) [24]. The most widely used strategy overcoming the instability is covering the carrier's with some hydrophilic polymers such as poly(ethylene glycol) (PEG) or poly(vinyl alcohol) (PVA). Nanocarriers linked with highly hydrated flexible PEG successfully escaped from the RES [25]. The PVA coating also improved the particle's stability. But as it should be a commonsense that introducing too much adjuvant into the body resulted in the undesirable toxicity. Moreover, the size, structure, and surface electronic properties of the formulations were changed resulting in unfavorable therapy index. On the contrary, the micellar system mainly including the polymeric micelle and phospholipid micelle has successfully overcome the above drawbacks because these spherical nanosized particles have simple structure and no adjuvant. The lipid based micelles show high potency in the doxorubicin entrapping [26]. But its intrinsic structure of phospholipid resulted in the untunable micellar structure with *D* > 100 nm, which considerably limited the intratumor accumulation. Additionally, drug release from conventional liposomal formulations is quite limited once these particles reach the tumor [27].

Fortunately, the nanosized polymeric micelles (10–100 nm in diameter) self-assembled from amphiphilic block copolymers can significantly improve the hydrophobic drug solubility in the core via the similar-to-similar interaction. The micelle possesses well defined hydrophobic core and hydrophilic corona structure in aqueous media [28]. On the other hand, the densely packed corona forming hydrophilic polymer chain can protect micellar system from the RES by reducing the interaction with serum proteins and renal filtration [29]. In comparison with lipid-based micelles, block copolymeric micelles provide a unique and powerful nanoplatform for anticancer drug delivery. The size of polymeric micelles can be easily tuned by varying the block lengths of the amphiphilic copolymer. It is also easy to modify micellar surface via the functional shell forming polymer. Both the tunable size range and the tailorable structure successfully reduce the renal filtration and obviously enhance tumor penetration. Some nanosized micelles such as PEG-PLA/PCL or PEG-PPO-PEG have significantly improved the *in vitro*/*vivo* application. Several polymeric micellar formulations are currently undergoing phase I/II clinical trials, which have shown significant antitumor efficacy and reduced systemic toxicity [20, 29, 30].

It is known that the endothelial cells of the tumor blood vessels proliferate at a 30–40-fold higher rate than those in normal tissues, which results in the larger endothelial cells gaps (200–700 nm, or sometimes even larger, up to 1.2 *μ*m) than 7 nm in the normal tissue [31]. Additionally, the high metabolism of tumor cells requires much more oxygen, nutrients, gas exchange, and waste removal. But the heterogeneity structure and distribution of the tumor blood vessels as well as the blood capillaries slow down the energy exchange between intra- and extratumor. All these result in unique characteristics of tumor, that is, the unnormal tumor blood vessels with gap in 200–700 nm [31], the relative high temperature of tumor (*T* > 37°C) [32], and the relative low pH (5~6) [31]. In order to further improve micellar delivering profile including the lesion's accumulating, cellular uptake, and intracellular release, many new stimulate-responsive micelles were extensively investigated with special focus on the tumor microenvironment. Utilizing the lower pH value in solid tumors and endosomes (5.5), Kataoka's group explored the novel multifunctional pH-sensitive doxorubicin-conjugated PEG-p(Asp-Hyd-DOX) copolymer micelles. The pH linker broke as pH < 6.0 ensued a sustain release [33]. An enhanced accumulation in lung and colon tumors of the micelle-forming PEO-PAsp (ADR) conjugates after 24 h (ca. 10% dose per g tumor) was much higher than the free ADR (ca. 0.90% dose per g tumor). Later, they further investigated the pH triggered intracellular release profile of poly(ethylene glycol)-poly(aspartate hydrazone adriamycin) micelles and observed that the micelles can stably circulated in physiological conditions (pH 7.4) and selectively release drug by sensing the intracellular low pH (pH 5-6). *In vitro* and *in vivo* studies show that the micelles had a good pH-triggered drug release capability, tumor-infiltrating permeability, and effective antitumor activity with extremely low toxicity [33, 34]. Okano's group used the temperature sensitive poly(N-isopropylacrylamide) (PNIPAM) to investigate the cellular uptake of bovine carotid endothelial cells [27]. As *T* > LCST, the cell uptake was significantly enhanced. In addition, the LCST of such PNIAPM can be tuned to *T* ~ 39°C by introducing some hydrophilic monomer into the chain backbone. Thus, the system can shabbily circulate at 37°C but be disassociated as *T* approaching to 39°C. This PNIPAM was also used to enhance the intracellular release because the cargo structure was disrupted as phase transition [35, 36]. The oxidative condition in the extracellular medium and reductive conditions in the tumor was used to enhance intracellular release. For example, the bioreducible PEG-SS-P[Asp(DET)] micelles bearing the disulfide bridge showed both 1–3 orders of magnitude higher gene transfection efficiency and a more rapid onset of plasmid DNA release than micelles without disulfide linkages [37]. Feng's group recently developed a micellar system containing a functional polymer of d-*α*-tocopheryl polyethylene glycol succinate (Vitamin E TPGS or TPGS), which stabilized the micelle and further promotes synergistic effects with the encapsulated drug [38]. This is a novel micellar system. The formulation formed by folic acid-conjugated d-*α*-tocopheryl polyethylene glycol succinate 2000 (Vitamin E TPGS2k) micelles successfully suppress the tumor cell growth [39]. For improving the therapeutic effect, some other intelligent micellar systems such as light responsive poly(methacrylate) and poly(acrylic acid) (PAzoMA-PAA) micelle were developed. This *trans-cis* photoisomerization of azobenzene group improved drug release [40]. In addition, the polymeric micelles conjugated tumor targeting *a*_*v*_ *b*_3_ ligand cyclic-(arginine-glycine-aspartic acid-d-phenylalanine-lysine) (cRGDfK) to DOXO-loaded polyethyleneglycol-polycaprolatone (PEG-PCL) micelles greatly enhanced internalization of the micelles through receptor-mediated endocytosis [41].

**Figure 1:**
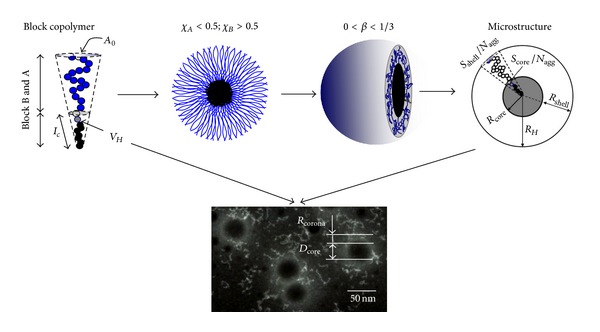
Finely self-assembly block copolymer micelles from the corresponding copolymers. The microstructure of such micelles and their electronic microscopy was also finely tailored [2].

These significant advances in intelligent block copolymer micelles have dawned upon a new era for nanomedicine. However, for translating an optimal micelle to clinical practice, there is still a big gap between *in vitro* and *in vivo* for lacking of understanding of the correlation between tumor unique characteristics (needs) and micellar physical chemistry properties (seeds). It is helpful to know that the micellar *in vitro/vivo* performance is strongly affected by its physical chemistry properties such as composition, dimension, microstructure, and the intelligent properties. The driving force for self-assembly is the strict solubility difference between the hydrophobic and hydrophilic blocks as described by the Flory-Huggins parameter (*χ*_*ps*_) [42]:

(1) $$\begin{array}{c} \chi_{\text{polymer},\;\text{solvent}}=\frac{{\left(\delta_{\text{polymer}}-\delta_{\text{solvent}}\right)}^{2}v_{s}}{KT}+0.34, \end{array}$$

where *δ*_polymer_ and *δ*_solvent_ are the solubility parameter of the polymer and solvent, *V*_*s*_ is the molar volume of solvent, *K* is Boltzmann constant, *T* is the temperature, and the value of 0.34 is entropic contribution, respectively. In fact, this force also determines the drug loading, that is, the interaction between drugs and core-forming polymer segment (*N*_segment_). The thermal translational energy per macromolecular is of the order of *k*_*B*_ *T*, whereas the interaction energy per macromolecules is proportional to its segment number *N*, namely, the product *N*_segment_ *χ*_polymer-drug_. This indicated that the micellar self-assembly and drug loading is directly related to the corresponding block copolymer composition. In addition, in aqueous solutions, the condition block copolymeric aggregated morphology was determined by the packing parameter *β*, which can be calculated by the following function (2):

(2) $$\begin{array}{c} \beta =\frac{V_{H}}{L_{C}A_{0}}, \end{array}$$

where the *V*_*H*_, *L*_*C*_, and *A*_0_ are the volume occupied by the hydrophobic chain, the hydrophobic chain counter length, and the surface area of hydrophilic chain, respectively. The condition for spherical micelle is 0 < *β* < 1/3. The correlation describing the assembly was illustrated in Figure 1. Thus, the block copolymer composition further determines the micellar size and structure. In our studies, it was found that the overall size (*D*) was related to the length of the amphiphilic block lengths by a scaling relation as *D* ∝ *N*_hydrophobic_^0.16^ *N*_hydrophilic_^0.6^. The micellar core/corona size (*D*_core_/*D*_corona_) and the drug loading into micelle (determined by the volume of core *V*_core_) were easily tuned by regulating core/corona forming block length [42]. On the other hand, as administrated to the body, both the extremely diluting (5 mL in one intravenous injection to 3500 mL blood circulated in human body) and the high shearing stress in viscostic blood stream (3.0 ~ 5.1 of whole blood viscosity > 1.0 of water) can deform the micelles. So its critical micelle concentration (CMC) should be as low as possible for avoiding* in vivo *disassociation. Additionally, micelle should also escape from the serum proteins absorption and removal by RES. In the experiment, we can tune the CMC by changing amphiphilic block lengths [43]. Moreover, it was found that decrease of the shell chain density (micellar surface area to aggregation number, *S*_corona_/*N*_agg_) strongly enhanced its stability. Increase hydrophobic/hydrophilic block length ratio resulted in *S*_corona_/*N*_agg_ decrease. Such entropic loss dominated the noncharged micellar *in vivo* escaping [43].

Based on the above-mentioned fundamental correlations, we further finely tailored *T* and pH sensitive poly(N-isopropylacrylamide-co-N,N-dimethylacrylamide-b-lacitde) and poly(N-isopropylacrylamide-*co*-*N,N*-dimethylacrylamide-*b*-*ε*-caprolactone) (PID_118_-b-PLA_59_ and PID_118_-b-PCL_60_) block copolymer micelles for enhancing tumor uptake and intracellular drug release [42]. The drug transported by these micelles was about 4 times higher than that by the commercial drug formulation, Taxotere. Both cytotoxicity assay against N-87 stomach cancer cell and confocal laser scanning microscopy (CLSM) confirmed the better transfection efficiency [42]. On the other hand, it is well known that the specifically targeting modifications can promote tumor accumulation. The targeting moieties such as antibody, folic acid, transferrin, and peptide (RGD) were used to decorate the particle surface. The targeting decorated nanocarriers can promote the binding with receptors on the cellular surface. Among the targeting molecules, the antibody represents a desirable moiety for its high specifically attaching ability. Recently, some novel antibody conjugating strategies are being developed in our group to enhance tumor accumulation by changing the binding site on the mAbs [44]. In short, to make more reliable block copolymer micelles for nanomedicine, we should firstly seek the major questions from the clinical oncology. Then it is essential to revisit and disclose the fundamental correlations including the inherent mechanism of micelle formation, effects of micellar properties on drug loading efficiency and releasing, *in vivo* stability, and tumor accumulation when we optimize high efficient next generation block copolymer micelles for cancer therapy.

## 3. Well-Defined Nanocarrier's Engineering for Immunotherapy

Various immune cells such as dendritic cells (DCs), B cells, and T-lymphocytes (TL) are recruited to the tumor. Modification of host immune system and/or utilization of components of the immune system for cancer treatment are called immunotherapy which mainly contains the active and passive form. Passive immunotherapy is to supply high amounts of effector molecules such as tumor-specific monoclonal antibodies (mAbs) to complement the immune system. Active immunotherapy is the utilization of humoral and/or cytotoxic T-cell effector mechanisms of the immune system following vaccination, namely, the cancer vaccines. This method can simultaneously activated antigen presenting cells (APCs), CD4+ T cells, CD8+ T cells, B cells, and innate immune cells, for example, granulocytes and NK cells. DCs are the most specialized and important APCs which are responsible for an adaptive immune response [45]. Vaccines based on lipid-based nanocarriers cannot only promote the accumulation in DCs in tumor-bearing hosts but also has a profound effect on DC function [46]. Poly(d,l-lactic acid-co-glycolic acid) (PLGA) nanoparticles carrying cancer-associated antigen (MUC1 mucin peptide: BLP25) and mouse specific peripheral lymphocyte antigen (MPLA) obviously promoted native T-cell activation in normal and MUC1-transgenic mice [47]. The efficiency of vaccination strongly depends on tumor specific antigens (TSAs) and vaccine delivery system. Polymeric nanoparticles attract extensive interest due to their facilely tunable composition, tailorable structure, unique intelligent properties, and high potential in cancer immunotherapy (i.e., the nanoimmunotherapy).

**Figure 2:**
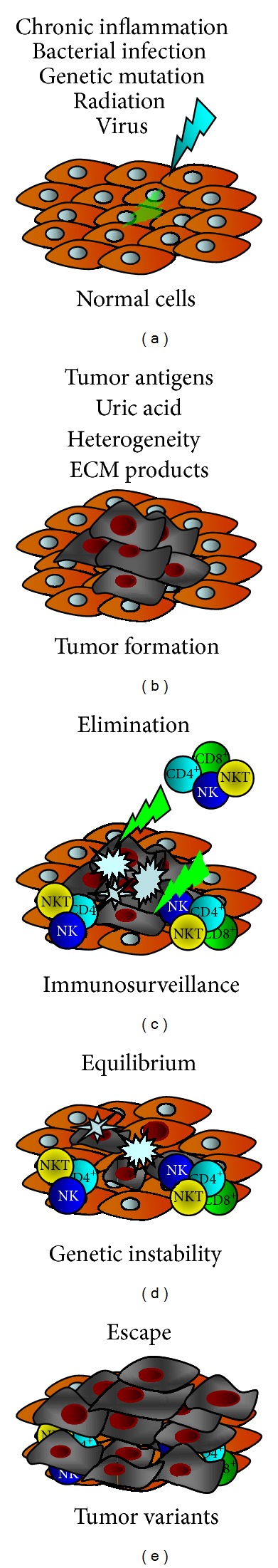
Scheme illustrates the tumor formation process ((a) and (b)) and smart tumor escape ((c)–(e)). ECM: extracellular level matrix.

The immunotherapy cannot only kill tumor cells in a specific manner but also alert the immune system to eradicate the disseminated tumor cells in blood circulation and micrometastases in distant organs [48, 49]. However, tumor cells can survive when they either maintain chronically or immunologically sculpt by immune “editors.” This well-known “immunoediting” refers to the elimination, equilibrium, and escape as illustrated by process (c), (d), and (e) in Figure 2. The new populations of tumor variants may eventually evade the immune system and escape from host immune surveillance by a variety of mechanisms including loss of MHC-I, adhesion molecules, tumor-associated antigens (TAAs), generation of regulatory T- (Treg-) lymphocyte, expansion of myeloid-derived suppressor cells (CD11b+ Gr-1+ cells, MDSCs), immunosuppression, blocking of NKG2D-mediated activation, and apoptosis induction of antitumor effector cells [50, 51]. Tumor-specific immune activation and nonspecific immune activation have been applied for overcoming such tumor escape. The tumor-specific immune responses are teaching the immune cells to recognize tumor cells specifically. B cells secrete antigen-specific antibodies which recognize, bind, and help to destroy the targets with the help from CD4+ T cells. CD4+ T cells recognize the antigens presented by MHC-II molecules and then stimulate B cells to produce antibodies to that specific antigen. Such antibody-coated cancer cells recognized and killed by NK cells, macrophage, and activated monocytes are called antibody-dependent cell-mediated cytotoxicity (ADCC). The nonspecific immune activation strategy mainly utilize the cytokines (IL2 and IL8), the interferons (IFN-*α*, *β*, and IFN-*γ*), and the Toll-like receptors (TLRs) for trigging DC maturation, stimulating proliferation of CD4+ and CD8+ T cells and modulating the suppressive function of regulatory T cells (Treg cells) [52]. Treg cells suppress TAA-specific immunity by inhibiting TAA-specific priming in tumor draining lymph nodes and further recruiting into the tumor microenvironment [53]. So depletion, blocking, and trafficking Treg-cell in tumors or reducing their differentiation and suppressive mechanisms represent new strategies for cancer treatment. It was known that knockdown of transcription factor Foxp3 gene in mature Treg cells resulted in the loss of their suppressive function [54]. However, the transfection efficiency is very low. But the newly developed novel carbon nanotubes (CNTs) can enhance Treg cells transfection [53]. The PLGA nanoparticle (PLGA-NP) carrying murine melanoma antigenic peptides hgp100_25-33_ and TRP2_180-188_ can also induce cytotoxic T lymphocyte responses against tumor-associated self-antigens in C57BL/6 mouse [55].

**Figure 3:**
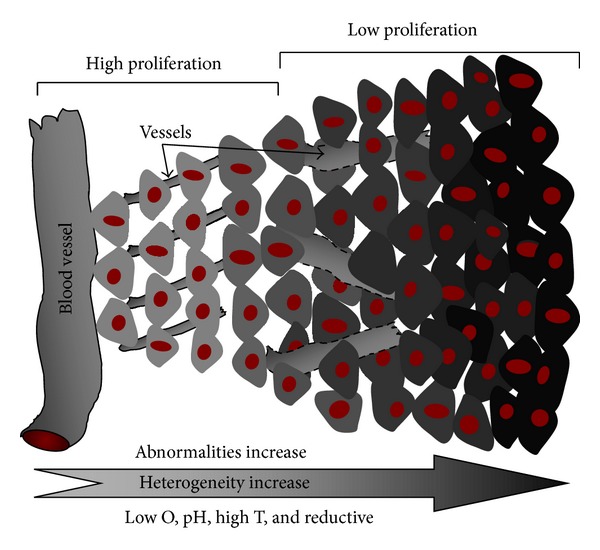
The general diagrammatic representation shows the abnormalities of cell proliferating profile and the blood vessels in solid tumors. With the depth (from the blood vessels) increase, the cell growth rate, the O_2_ concentration, and the pH decrease [2].

**Figure 4:**
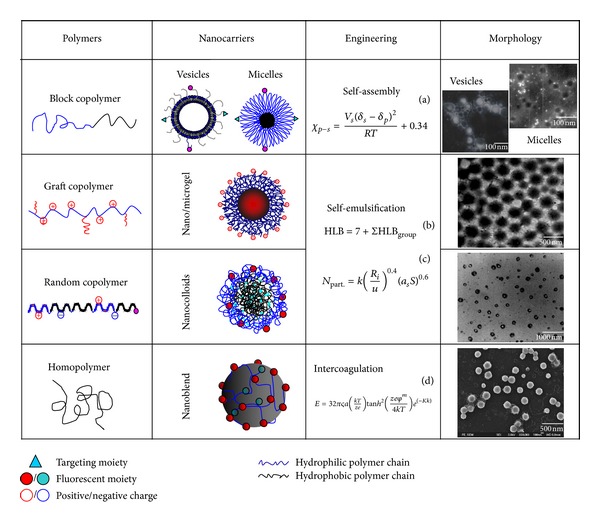
TEM images show the well-defined structure of functional nanocarriers engineered by tuning the monomer ratio, tailoring the polymer composition, and regulated by different particle's formation mechanisms. (a) The flory interaction parameter (*χ*), where *δ*_*p*_ and *δ*_*s*_ are the solubility parameter of the polymer and solvent, respectively, *V*_*s*_ is the molar volume of solvent, *K* is Boltzemann constant, and the value of 0.34 is entropic contribution; (b) the hydrophilic to lipophilic balance (HLB), where HLB_group_ is the constant of different groups along polymer chain; (c) the particle number calculation in emulsion (N_part._), where *k* is consistent in the range of 0.37–0.53, *R*_*i*_, *u*, *a*_*s*_, and *S* are the rate of total radicals produced, the rate of the particle volume increase, the surface area of a surfactant, and total number of surfactant, respectively; and (d) the electrostatic repulsion energy (*E*), where the *ς*, *k*, *z*, *e*, *φ* ^*m*^, *κ*, and *h* are the electronic constant of the solvent, Boltzmann's constant, the number of ion, the capacity of solvent, the double layer potential of the diffusion layer, the thickness of the double layer, and the distance between two particles, respectively.

Thus, finely engineering nanocarriers from homopolymers, copolymers, and lipids with high loading and transferring efficiency, site-specific targeting to immune cells, high *in vitro*/*vivo *stability, and intelligent responsive to tumor microenvironment shows high potent in nanoimmunotherapy [56, 57]. Tumor microenvironment is main battlefield for tumor escape and immune system activation. As shown in Figure 3, the high proliferation and metabolism of tumor endothelial cells resulted in the unique properties of tumor microenvironment including large endothelial cells gaps (200–1000 nm), the relative high temperature (*T* > 37°C), low pH (5~6), lacking of lymphatic nodes, and lymph vessels [4, 58]. This unique pathological condition of microenvironment offers challenges for novel nanocarrier's engineering. Based on the self-assembly mechanism, well-defined micelle and vesicle with surface targeting decorating were finely engineered (Figure 4) [4, 59]. We found that the temperature regulated passive and mAb tuned active dual targeting immunomicelles significantly enhanced intratumor accumulation and cellular uptake [4]. The nanostructure and dimension were also tailored to match the large endothelial cells gaps in tumors with enhanced permeability and retention (EPR) [47]. The extracellular pH is ~7.4, but the pH in the endosome and microenvironment is ~6.0. This value is still lowered to ~5.0 in the lysosome. The hydrolysis rates of polyester such as polylactic acid, polyglycolic acid, and their copolymers can thus be tuned for endosomal and/or lysosomal delivery [60]. Additionally, the endosome is reductive, but the lysosomal is oxidative. This difference is very important for spatial delivery antigens for MHC presentation. Because the antigens for MHC class I pathways must be available in cytosol whereas those for MHC class II molecules must be present in endolysosome. The finely engineered lipids with protein antigens in nanovesicle core and lipid-based immunostimulatory molecules in the walls successfully elicits endogenous T cell and antibody responses, which showed rapid release adjuvants in the presence of endolysosomal lipases [61]. Some danger signals (adjuvants) for APC activation are present on the plasma membrane. So nanocarriers engineered from polycations such as polyethyleneimine (PEI) or its graft copolymers (Figure 4) hold favorable effect on membrane destabilization by the “proton-sponge” effect which can also control the endosomal release [62]. Both structural defects and fibrosis of the interstitial matrix result in poor/dysfunctional T-cell priming in tumor microenvironment. But forced expression of the tumor-necrosis factor (TNF) can induce naive T-cell priming. Thus, delivery stimulator such as CD80, interleukin-4 (IL-4), and cytokines by intelligent nanocarriers to tumor microenvironment can produce T-cell priming with the microenvironment reversion [63].

DCs appear in most peripheral tissues where antigens typically first encounter the immune system. Immature DCs phagocytose the encountered antigens followed by the activation, maturation, and migration to draining lymph nodes. They present antigens to their cognate naive T-cell partners and instruct the anergy, tolerance, or immunity. Then the antigen specific T-cell immunity is initiated [45]. Noted here, timing at which antigen and adjuvant reach DCs is crucial. If the maturation stimulus is too late, tolerance will be induced. If the antigens reach mature DCs, they will not be efficiently presented. The intelligent responsive polymer carriers can be finely designed to regulate the antigen's communication with DCs. Some lipids had successfully been used to promote the lymphatic trafficking and endue the DCs mutation [64]. The DCs preferentially take up smaller particles with size similar to viral (~20 nm), whereas macrophages ingest the large particles with size around bacterial. It is also worth mentioning that PLGA-NPs (500 nm) are more effective than microparticles (~2 nm) in stimulating CTL responses. The DC's phagocytosis is also affected by nano/microparticle's surface charge [65]. Cationic particles are particularly effective for uptake by DCs and macrophages due to that the ionic attraction increases the particle binding and internalization. As shown in Figure 4, above-mentioned nanocarrier's size, microstructure, charge, and intelligent properties can be facilely engineered by tuning polymer composition and particle formation process. In addition, specific DC-specific antibodies such as anti-CD11c and anti-DEC205 can enhance nanocarrier's accumulation in DCs. The PLA nanoparticles loaded dacarbazine (DTIC) decorated with TRAIL-receptor 2 (DR5) mAb (DTIC-NPs-DR5) showed high internalization by DR5-overexpressing metastatic melanoma and chemo-immunocooperative therapeutic effects [66]. Benefit from our understanding of the molecular mechanism of immunoescape and the physiologic conditions of tumor, the nanocarriers in nanoimmunotherapy should be further finely engineered with well-defined dimension, intelligent properties, specific targeting, advance lymphatic imaging, and precisely intracellular release for optimizing the therapeutic index [2].
